# Chewing gum for intestinal function recovery after caesarean section: a systematic review and meta-analysis

**DOI:** 10.1186/s12884-017-1286-8

**Published:** 2017-04-18

**Authors:** Zunjia Wen, Meifen Shen, Chao Wu, Jianping Ding, Binbin Mei

**Affiliations:** 1 The First Affiliated Hospital of Soochow University, No. 188 Shizi Street, Gusu district, Su Zhou, Jiangsu province China; 2 Nursing School of Soochow University, Su Zhou, China

**Keywords:** Caesarean, Gum, Intestinal function, Meta-Analysis, Recovery, Review

## Abstract

**Background:**

Gum chewing has been reported to enhance the intestinal function recovery after caesarean section, current perspectives and practice guidelines vary widely on the use of gum chewing, more studies on the role of gum chewing after caesarean section are needed.

**Methods:**

We performed a comprehensive, systematic meta-analysis of randomized controlled trials (RCTs) on the efficacy of gum chewing after caesarean section. Studies were identified by searching EMBASE et al database (until June 30, 2016). Summary odd ratios or weighted mean differences with 95% confidence intervals were calculated for each outcome with fixed- or random-effects model.

**Results:**

Ten RCTs with a total of 1659 women were included in our meta-analysis. Gum chewing provided significant benefits in reducing the time to first passage of flatus, first defecation, first bowel sound, first bowel movement and the length of hospital stay, but not in the time to first feeling of hunger.

**Conclusions:**

Gun chewing hastens the intestinal function recovery after caesarean section and offers a safe and inexpensive option. High-quality and larger-scale RCTs are still warranted to clarify the role of gum chewing in intestinal function recovery after caesarean section.

## Background

With the development of medical care and policy supports, the caesarean delivery, a most commonly seen operation, has increased worldwide over the past decades [1]. However, it may lead to many complications such as postoperative ileus with a mean incidence of 10–15% [2, 3], result in longer hospital stay, increased postoperative morbidity and excessive medical costs [4].

Promoting intestinal function recovery after caesarean section is on the top of research agenda of healthcare providers. Traditionally, physicians forbid oral feeding with concern to the risks of intestinal fistula, re-bleeding and aspiration mistakenly [5], but recent studies have supported that early postoperative feeding can stimulate bowel motility and shorten hospital stay [6, 7], however, patients may not tolerate the early feeding regimen [8]. Several methods have been proposed to accelerate the return of gastrointestinal motility after caesarean section, including early oral hydration, ambulation and gum chewing.

Gum chewing as a kind of sham feeding was introduced in hope that it may hasten the intestinal function recovery in recent years, by means of stimulating the cephalic vagal reflex, the hormones secretion may increase [9]. The efficacy of gum chewing have been fully elucidated in the intestinal function recovery in patients after colorectal surgery and it can provide various benefits [10, 11], yet the efficacy of chewing gum after caesarean section remain inconsistent. Several previous meta-analysis [12–17] including randomized controlled trials (RCTs) on the gum using after caesarean have been conducted, but the included data are rather limited. Up to date, no guideline has officially support the use of chewing gum for intestinal function recovery in obstetrics and gynaecology, more evidences of higher quality on this issue are warranted.

Given the recently emerging evidence on the role of gum chewing after caesarean section, we performed this systematic review and meta-analysis of RCTs with the following objectives: (1) to review and sum up the current evidence on the influence of gum chewing in intestinal function after caesarean section; (2) to compare the efficacy of chewing gum and standard care in intestinal function recovery after caesarean section; and (3) to analyze and conclude the appropriateness of gum chewing in patients after caesarean section.

## Methods

### Search strategy

We tried to plan, perform and report this meta-analysis in comply with PRISMA guideline [18]. Related articles either published in English or Chinese were identified and selected by searching PUBMED, EMBASE, Science Direct, Cochrane Central Register of Controlled Trials, China National Knowledge Infrastructure (CNKI) and Wanfang Database (until June 30, 2016) using the following search terms including “gum-chewing”, “chewing-gum”, “sham-feeding”, “‘caesarean section”’, “caesarean”, “caesarean delivery”, we combined these terms in accordance to the instructions of the database. In addition, the reference lists of the retrieved studies and pervious reviews and meta-analyses were reviewed and manually searched, and we made no attempt to identify unpublished reports.

### Study selection

Study selection was made based on a first screen of identified titles or abstracts and on a second check-up of full-text articles. Studies were considered to be eligible if the criteria below were met: (1) RCT design; (2) study subjects had receiving caesarean section; (3) included the comparison groups of added gum chewing and standard nursing care post caesarean delivery; and (4) the relative outcome data (the time to first passage of flatus after operation, et al) were reported. Studies were excluded if: (1) it’s not RCT design, which may produce more heterogeneity for data analysis; (2)the study subjects had not received caesarean section; (3) the relative outcome data were not accessible for data synthesis.

### Data extraction

The following data information were extracted by two reviewers independently: first author, year of publication, country, patient population, participants, methods of gum chewing, other concurrent interventions, main outcomes and study results. The studies selection and data extraction were conducted by two authors independently, any disagreement was resolved by further discussion.

The outcomes we collected for data analysis included: (1) the time to first passage of flatus; (2) the time to first defecation; (3) the time to first bowel movement; (4) the length of hospital stay; (5) time to first feeling of hunger; (6) the time to first bowel sound; (7)postoperative complications such as nausea, abdominal distension, vomiting et al.

### Assessment of the quality of the included studies

The Cochrane Collaboration’s “risk of bias” tool was adopted for evaluating the methodological quality and risk of bias of included RCTs, seven specific domains were examined and measured in this tool: sequence generation, allocation concealment, blinding of participants and personnel, blinding of outcome assessment, incomplete outcome data, selective outcome reporting, and “other” issues. Every domain could be classified as “low risk of bias”, “high risk of bias” or “unclear risk of bias” under the guidance of judgment criteria (Cochrane Handbook for Systematic Reviews of Intervention. Part 2: 8.5).

### Data synthesis and analysis

All the extracted data were processed in a freeware program Review Manager (RevMan) Version 5.3. Binary outcomes (i.e., VAP and mortality) were presented as Mantel-Haenszel style odd ratios (ORs) with 95% confidence intervals, and continuous outcomes were reported as inverse variance weighted mean differences (WMDs). A fixed-effect model was adopted in cases of homogeneity (*p* value of *χ*2 test >0.10 and I^2^ < 50%), while a random-effects model was used in cases of obvious heterogeneity (*p* value of *χ*2 test >0.10 and I^2^ > 50%). Publication bias was evaluated by the demonstration of funnel plots, and asymmetry was assessed by means of the Egger regression test (*p*-value < 0.1 was considered to be significant of funnel plot asymmetry).

## Results

### Literature search

**Fig. 1:**
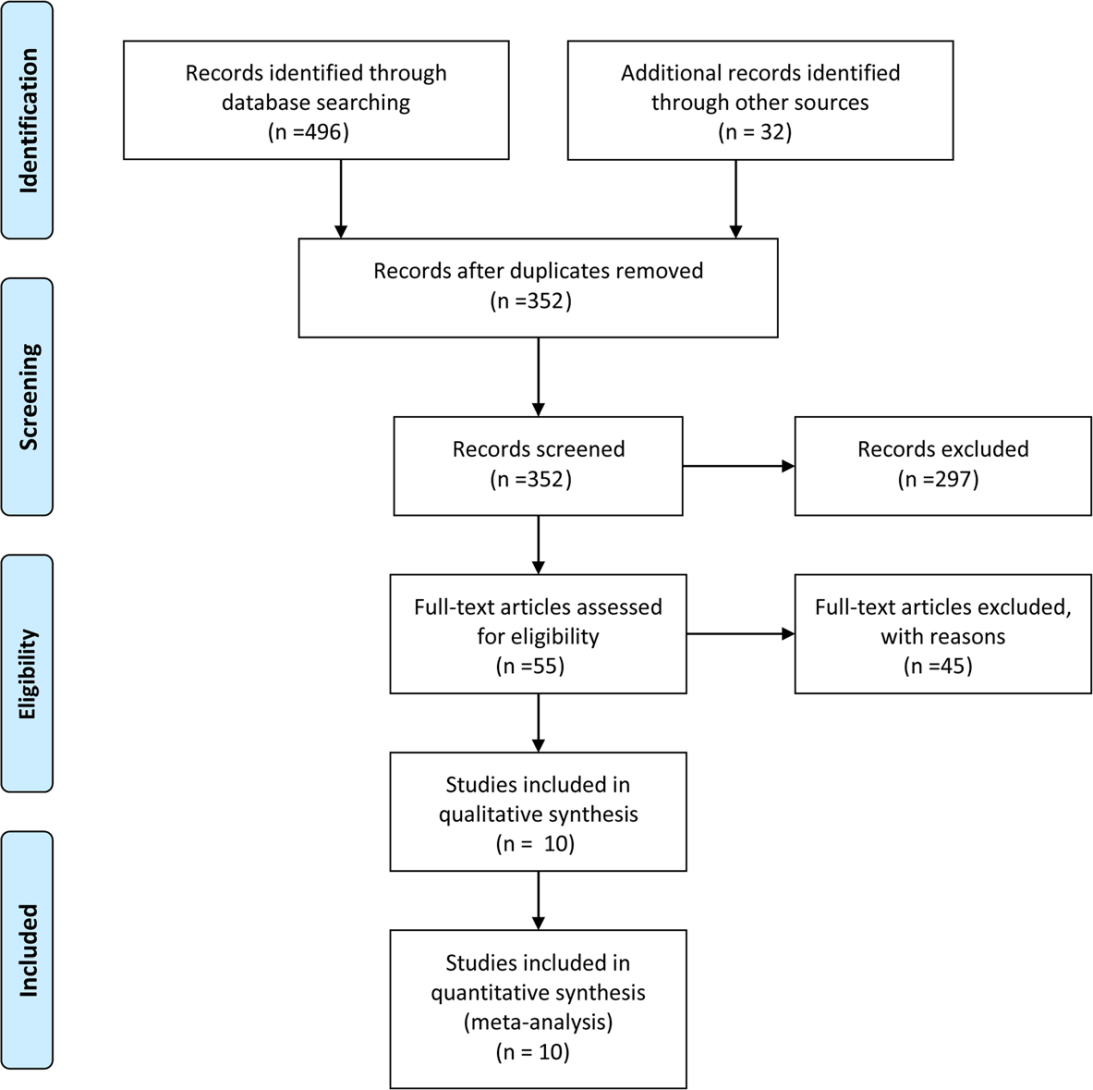
Flow diagram of study selection

A sum of 528 relevant publications were yielded by the comprehensive search, and the first screen of titles and abstracts excluded 473 papers, and 55 potentially related studies were included for further full-text review, participants and interventions not meeting the including criteria were the main concern for further consideration, eventually, 10 RCTs [19–28] were included for data analyses (Fig. 1).

### Study characteristics

The basic characteristics of 10 included studies are shown in Table 1. In brief, a total of 1659 patients were involved, specifically with 835 patients for gum chewing intervention, and 824 for standard postoperative care, the countries of included studies ranged from Egypt, Nigeria, Thailand, Turkey, Iran, Philippines to China, and four RCTs were conducted in China, the numbers of included participants among studies differed from 50 to 386. It’s noteworthy that one study was reported as conference paper, although the structure and data were relatively complete, the details on how study conduct remain unclear, we attempted to contact the original authors for more information, yet no response was achieved, therefore, we still included this study for data analysis, but we put more cautions on the quality of study details. All studies have reported no statistical significant differences in the baseline characteristics between intervention and control groups. Meanwhile, some differences among studies were found in the gum chewing interventions, including the timing of initiation and frequency of gum chewing, one study [24] also compared the efficacy of xylitol-containing and xylitol-free gum chewing with standard postoperative care protocol, in our study we only extracted the data in xylitol-free gum chewing and control groups. Interestingly, all the results favored the usage of gum chewing after caesarean sections.

**Table 1:** The characteristics of included studies

Author(year)	Country	Numbers of participants(GC/NGC)	Gum intervention	Main outcomes	Results
Abd-El-Maeboud 2009 [19]	Egypt	200(93/107)	Started from 2 h after surgery, lasted for 15 min every 2 h in the daytime but not in the night, until the passage of flatus	①,②,④,⑤	Gum chewing after CS is safe, well tolerated, and associated with rapid resumption of intestinal motility and shorter hospital stay
Ajuzieogu 2014 [20]	Nigeria	180(90/90)	Started from the first day after operation for 5 consecutive days, 3 times daily, 30 min for every time.	①,②,④,⑤	Gum chewing has a beneficial effect on early return of bowel function following cesarean section and should be included in the postoperative management protocol
Jakkaew 2013 [21]	Thailand	50(25/25)	Started since the regain of consciousness after operation, 4 times a day (morning, noon, evening, and before bed time), 30 min for every time, until the first passage of flatus	①,⑤,⑥,⑦	Gum chewing is associated with faster recovery of bowel function following cesarean section
Kafali 2010 [22]	Turkey	150(74/76)	Started from 2 h after surgery, 3 times daily in the morning, afternoon, and evening, each episode of gum chewing lasted 1 h except the initial one which lasted 15 min	①,④,⑤	Gum chewing provides a simple method for early recovery of bowel function after cesarean section
Ledari 2012 [23]	Iran	100(50/50)	Chewing gum for at least 1 h, three times daily from 6 h after surgery (after recovery from anesthesia) until being discharged	①,④,⑥	chewing is acceptable and inexpensive physiologic method for decreasing the time to the passage of flatus, bowel movements, and feeling of hunger in patients undergoing cesarean section
Lee 2016 [24]	China	120(40/40/40)	Chewing xylitol-free or xylitol-containing gum started from 2 h after surgery, repeated every 2 h between 9 a.m. and 8 p.m, 15 min for every time, until the first passage of flatus	①,②,④	After cesarean section, chewing gum increased participants’ return of bowel activity and xylitol-containing gum may be superior to xylitol-free gum
Liang 2007 [25]	China	120(60/60)	Started after recovery from anesthesia, repeated every 2 h for 15 min every time	①,②,⑦,⑧	Chewing gum can promote gastrointestinal motility recovery after cesarean section, and the method is simple, convenient, safe
Luo 2010 [26]	China	300(150/150)	Started from 2 h after surgery, 4 times daily in the morning, afternoon, and evening, each episode of gum chewing lasted 10-15min	①,②,④,⑦,⑨	Chewing gum can promote the recovery of gastrointestinal function after cesarean section
Shang 2010 [27]	China	386(195/191)	Chew gum for at least half an hour, three times per day after returning to the ward	①,②,④,⑤,⑩	Gum chewing is an inexpensive, convenient, and physiological method in enhancing the recovery of bowel function
Zamora 2012 [28]	Philippines	53(18/35)	Gum chewing involved chewing two pellets of sugarless gum at 12 h post operation for 15 min then advanced to sips of clear liquids at 16 h post operation	①,③,⑤,⑩	Postoperative gum chewing stimulates the earlier return of bowel motility after cesarean delivery

*Notes*: ①, the time to first passage of flatus; ②, the time to first defecation; ③, the time to first bowel movement; ④, the time to first bowel sound; ⑤, the length of hospital stay; ⑥, time to first feeling of hunger; ⑦, abdominal distension; ⑧, vomiting; ⑨, post operation incision pain; ⑩, postoperative ileus(POI)

### Methodological quality and risk of bias

**Fig. 2:**
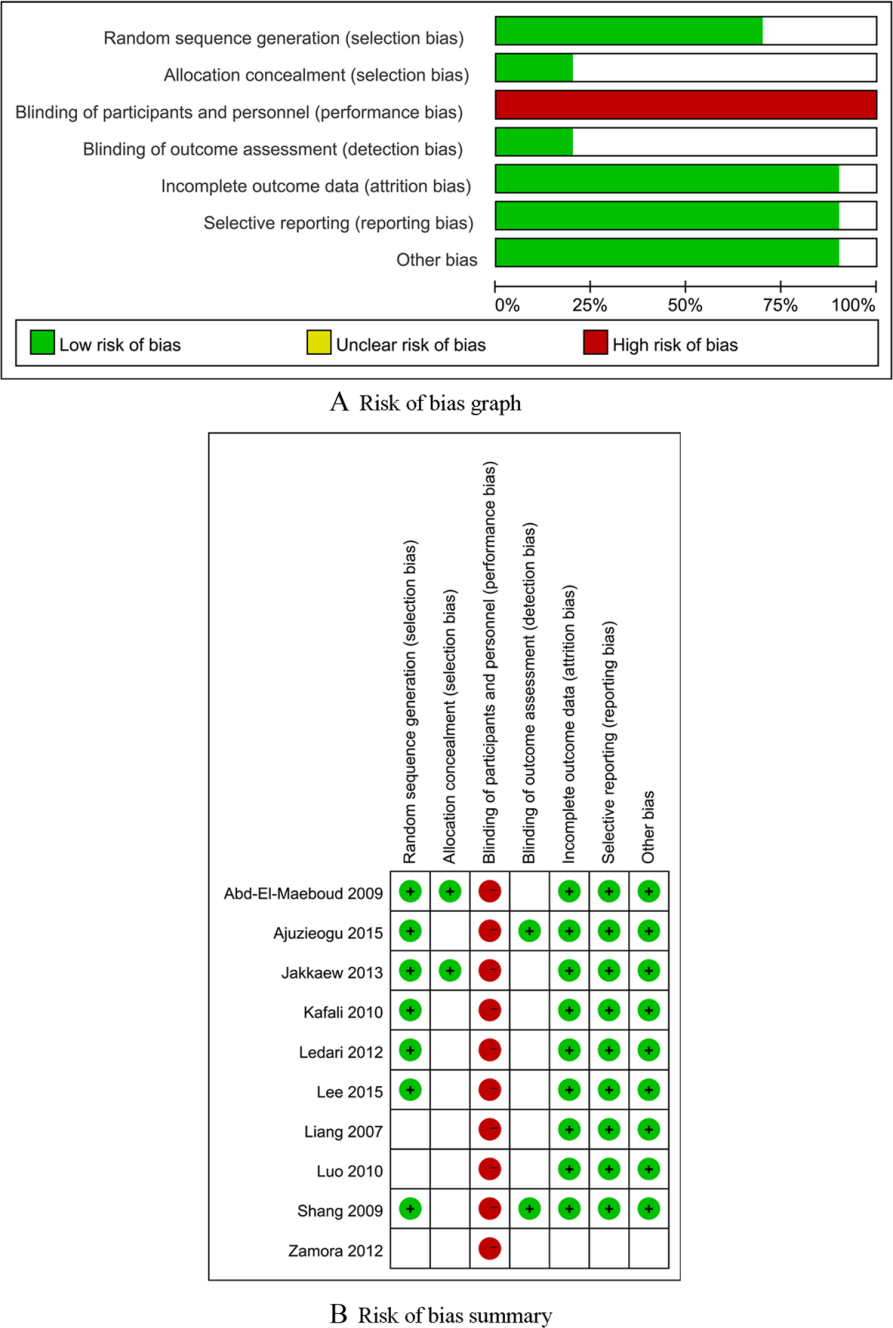
Methodological quality and risk of bias of the included studies

Figure 2a illustrates all of the bias classifications in a way of cross-tabulation for the included RCTs, and the summary of qualitative methodological quality abiding by the bias classification (“Low risk”, “High risk”, “Unclear risk”) is presented in Fig. 2b. Briefly, all included RCTs mentioned that the randomization was adopted in their studies, yet three RCTs [25, 26, 28] made no reports on the methods for random sequence generation, only two studies reported the strategies to achieve allocation concealment and then were marked as low bias of risk. Considering that it’s hard to blind patients and care providers on the gum chewing intervention, most RCTs adopted single-blind or no blind study design instead of a double-blind study design, all RCTs made no blind study design on participants and personnel then were classified as high risks of bias, and only two [19, 27] adopted the blind design on outcome assessment. Except for the details lack of Zaroma’ study [28],all the other RCTs have provided the necessary data and outcomes for selective reporting and other bias evaluations.

### Main analysis

**Fig. 3:**
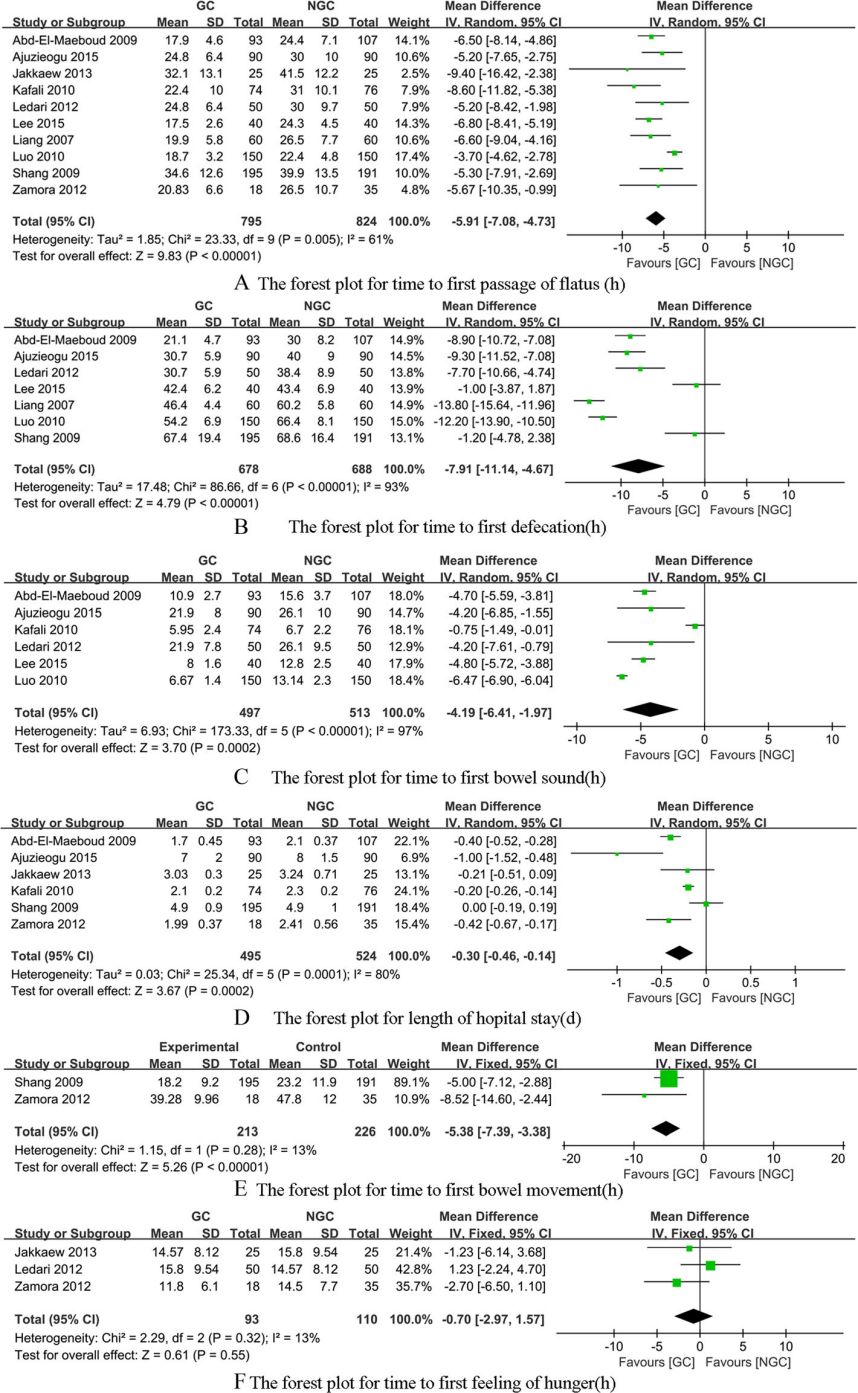
The forest plots for different outcomes

The WMDs for each study on the time to first passage of flatus, the time to first defecation, the time to first bowel sound, the length of hospital stay, the time to first bowel movement, time to first feeling of hunger are shown in Fig. 3.

*The time to first passage of flatus* All included studies have reported the time to the first passage of flatus, The summary WMDs on the time to first passage of flatus (hours) was -5.91(95% CI: -7.08– -4.73), with evidence of heterogeneity (*P* = 0.005, I^2^ = 61%) (Fig. 3a).

*The time to first defecation* Seven studies [19, 20, 23–27] have reported the time to the first passage of flatus, The summary WMDs on the time to first defecation (hours) was -7.91(95% CI: -11.14– -4.67), with evidence of heterogeneity (*P* < 0.001, I^2^ = 93%) (Fig. 3b).

*The time to first bowel sound* Six studies [19, 20, 22–24, 26] have reported the time to first bowel sound, The summary WMDs on the time to first bowel sound (hours) was -4.19(95% CI: -6.41– -1.97), with evidence of heterogeneity (*P* < 0.001, I^2^ = 97%) (Fig. 3c).

*The length of hospital stay* Six studies [19–22, 27, 28] have reported the length of hospital stay, The summary WMDs on the length of hospital stay (days) was -0.30(95% CI: -0.46– -0.14), with evidence of heterogeneity (*P* = 0.28, I^2^ = 80%) (Fig. 3d).

*The time to first bowel movement* Two studies [27, 28] have reported the time to first bowel movement, The summary WMDs on the time to first bowel movement(hours) was -5.38(95% CI: -7.39– -3.38), with no evidence of heterogeneity (*P* < 0.001, I^2^ = 13%) (Fig. 3e).

*Time to first feeling of hunger* Three studies [21, 23, 28] have reported time to first feeling of hunger, The summary WMDs on time to first feeling of hunger (hours) was -0.70(95% CI: -2.97– 1.57), with no evidence of heterogeneity (*P* = 0.32, I^2^ = 13%) (Fig. 3f).

### Publication bias analysis

**Fig. 4:**
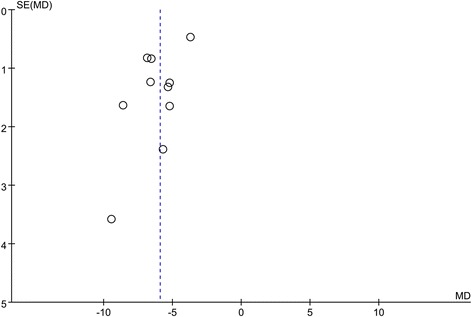
The funnel plot for the time to first passage of flatus

Funnel plot, a simple scatter plot of the intervention effect estimates from every studies, is plotted against some measure of each study’s size or precision (Cochrane Handbook for Systematic Reviews of Intervention. Part 2:8.5). Ten or more studies are required for the significant evidence of funnel plot. In our studies, due to the limitation of included data, we only performed funnel plot analysis on the time to first passage of flatus. As presented in Fig. 4, the distribution of study estimates in the funnel plot was symmetrical and well-proportioned, no significant publication bias have been found.

### Subgroup and sensitivity analyses

No subgroup analyses were made in this study. Sensitivity analyses which investigate the impact of a single study on the overall risk estimate by abandoning one study in each turn. In the process of sensitivity analyses, we found that the results of time to first bowel movement changed when we neglected the study conducted by Shang [27], yet the data on this outcome still quite limiting. For other outcomes, no significant change of the overall risk estimates by abandoning any single study was found.

## Discussion

The results of our meta-analysis have indicated gum chewing after caesarean section can significantly promote the intestinal function recovery by accelerating the time to first passage of flatus, first defecation, first bowel sound, first bowel movement and shortening the length of hospital stay. The gum-chewing groups’ faster recovery of gastrointestinal tract motility may result from stimulating intestinal motility by strengthening the cephalic-vagal reflex and increasing the gastrointestinal hormones secretion associated with bowel motility [9]. However, we did not find benefits of gum chewing on the time to first feeling of hunger, one possible explanation is that the chewing movement cause a feeding feedback to the brain, making a full feeling for our central nerve systems, additionally, the early feeding program employed in some studies can further enhance the hunger stimulus, previous study [10] has concluded that sham feeding confers no advantage if patients are placed on a rapid postoperative feeding regimen. Also, all the included studies have reported no side effects of gum chewing intervention in patients after caesarean section, therefore, gum chewing may be a safe and feasible intervention for accelerating the intestinal function recovery in patients after caesarean section.

Similar to other meta-analyses, our study has upheld the use of gum chewing in the early stage after caesarean section to accelerate the intestinal function recovery. A recent meta-analysis by Huang [12] included only five RCTs involving 882 patients, the sample size and number of included studies were small. An earlier meta-analysis by Hochner [15] also included five RCTs with a total of 846 participants, the process for meta-analysis was rather strict, yet the publication bias could not be evaluated quantitatively by a funnel plot due to the small number of studies available, the other meta-analyses [14, 16, 17] also had problems in this area. Another comprehensive Cochrane meta-analysis [13] identified 81 studies involving 9072 participants to investigate whether the chewing gum after surgery hasten the return of gastrointestinal function after abdominal surgery, come to the finding that gum chewing showed a beneficial impact on the major outcomes of digestive system activation, including bowel sound, flatus passage and bowel movement, yet the including studies were rather heterogeneous. Different from previous meta-analysis, with more related RCTs published, our study has included more RCTs for synthesized analysis, which may have more advantages in reducing publication bias.

Several previous meta-analyses [10, 29] focused on the influence of chewing gum after colorectal cancer surgery, come the finding that it might promote the recovery of intestinal function. A meta-analysis [30] including12 RCTs focused on chewing gum and postoperative ileus in adults, found that chewing gum provided small benefit in reducing time to flatus, and time to bowel motion, but not in the length of stay or the incidence of complications, it’s noteworthy that this studies included patients receiving either colorectal surgery or cesarean sections, in which lots of heterogeneities might exist, it should be emphasized that the surgery differences may lead to different injuries to the intestinal function, and gum chewing may offer different effects on the recovery process, specific intervention details(starting time, frequency et al.) of gum chewing for different surgeries remain further investigation.

Although early oral intake has been promoted as standard care after caesarean section [31], only one RCT [22] adopted oral intake within six hours postpartum in both groups, still, when early oral intake was standardized in each group, the gum chewing added more benefits to the intestinal function recovery. A recent study [32] compared the effect of gum chewing, early oral hydration, and early mobilization on intestinal motility after cesarean birth, concluded that all of the three different interventions increased intestinal motility, and should be recommended during postoperative routine care to shorten hospital stay and prevent postoperative ileus, future research needs to further clarify the roles of those three interventions. Notably, one included RCT [24] also evaluated the effects of xylitol-containing and xylitol-free gum chewing on gastrointestinal recovery after cesarean section, the results indicated that xylitol-containing gum might be superior to xylitol-free gum, while the most included RCTs adopted the sugar-free gum in their interventions. The sugar ingredients in sugar free gum (e.g., sorbitol and xylitol) may stimulate bowel mobility and exert a laxative effect, yet the evidence on whether sugar substitutes in sugar-free gum affect bowel function remain conflicting [33, 34], more researches in this area are also warranted.

Several limitations in this meta-analysis should be considered. First, there are some differences in gum chewing interventions (e.g., initiation, ingredients and frequency) among included RCTs, this is most likely accountable for the heterogeneity observed, however, due to the data limitations, we did not perform meta-regression analyses on patient population characteristics. Second, the quality of the included studies was not rather high, most included RCTs made no report on the randomization concealment and blinding, due to the nature of gum chew intervention, it’s rather difficult to blind the participants in this study setting, but blinding the observers is achievable and might reduce the bias, future studies should put more attentions on the RCT design. Third, limited by study data, we did not perform subgroup analysis and funnel plot in some outcomes, still, it’s possible that the end point susceptible to missing data from studies that were not published due to an overall null effect.

## Conclusions

In conclusion, our study has further supported that gum chewing is associated with early recovery of intestinal function after caesarean section, which may be helpful to reduce the time to first passage of flatus, first defecation, first bowel sound, first bowel movement and shorten the length of hospital stay. Gum chewing offers a safe, simple and inexpensive for hastening the recovery of intestinal function after caesarean section, which is worthy of promotion for clinical use.

## Abbreviations

CNKI: China National Knowledge Infrastructure
ORs: Odd ratios
RCTs: Randomized controlled trials
WMDs: Weighted mean differences
